# Using Novel Implementation Tools for Evidence-based Intervention Delivery (UNITED) across public service systems for three evidence-based autism interventions in under-resourced communities: study protocol

**DOI:** 10.1186/s12888-022-04105-9

**Published:** 2022-07-16

**Authors:** Jill Locke, Elizabeth McGhee Hassrick, Aubyn C. Stahmer, Suzannah Iadarola, Brian Boyd, David S. Mandell, Wendy Shih, Lisa Hund, Connie Kasari, AIR-B Network

**Affiliations:** 1 Department of Psychiatry and Behavioral Sciences, University of Washington, 6200 NE 74th St, Bldg. 29, St. 100, Box 357920, Seattle, WA 98115 USA; 2 A J Drexel Autism Institute, Drexel University, 3020 Market Street, Philadelphia, 19104 USA; 3 Department of Psychiatry and Behavioral Sciences, University of California, 2825 50th Street, DavisSacramento, CA 95817 USA; 4 Department of Pediatrics, University of Rochester Medical Center, 601 Elmwood Ave, Box 671, Rochester, NY 14642 USA; 5 Juniper Gardens Children’s Project, University of Kansas, 444 Minnesota Ave., Ste 300, Kansas City, KS 66101 USA; 6 Department of Psychiatry, University of Pennsylvania Perelman School of Medicine, 3535 Market Street, 3rd floor, Philadelphia, PA 19104 USA; 7 Center for Autism Research & Treatment, Semel Institute, University of California, 760 Westwood Plaza, Los AngelesLos Angeles, CA 90024 USA; 8 Health Resources and Services Administration, Maternal and Child Health Bureau, Office of Epidemiology and Research, Division of Research, U.S. Department of Health and Human Services, 5600 Fishers Lane, Rockville, MD 20857 USA; 9 Human Development & Psychology, Graduate School of Education & Information Studies, Moore Hall 3132B 405 Hilgard Avenue, Los Angeles, CA 90095 USA

**Keywords:** Implementation strategy, Social network analysis, Collaborative teaming, Autism, Mind the gap, Remaking recess, Self-determined learning model of instruction, Stages of implementation completion

## Abstract

**Background:**

There are a growing number of evidence-based interventions (EBIs) for autistic individuals, but few are successfully implemented with fidelity in under-resourced communities and with families from traditionally disenfranchised groups. Implementation science offers tools to increase EBI use in communities, but most implementation strategies are designed specific to a single EBI. It is not feasible to develop a new implementation strategy each time a new EBI is introduced in the community. Therefore, to test the effectiveness and generalizability of implementation strategies we are developing and testing a multifaceted implementation strategy with three EBIs concurrently. The goal of this protocol paper is to describe the randomized field trial of an implementation strategy for use across autism EBIs, diverse settings and participants, with the goal of increasing rapid uptake of effective practices to reach our most vulnerable children.

**Methods:**

We developed a multifaceted implementation strategy called Using Novel Implementation Tools for Evidence-based intervention Delivery (UNITED) to facilitate the implementation and sustainment of three EBIs in under-resourced settings. We will compare fidelity to, and effectiveness of, each intervention [Mind the Gap (MTG), Remaking Recess (RR), Self-Determined Learning Model of Instruction (SDLMI)] with and without UNITED in a randomized field trial. Randomization will be stratified using a minimization allocation method. We will train community practitioners using remote delivery of modules specific to the intervention, and active coaching via Zoom for at least 6 sessions and up to 12 as dictated by each EBI. Our primary outcome is fidelity to each EBI, and our secondary outcome is at the child or family level (family empowerment for MTG, child peer social engagement for RR, and adolescent self-determination for SDLMI, respectively). We will measure progress through the implementation phases using the Stages of Implementation Completion and cost-effectiveness of UNITED.

**Discussion:**

The results of this study will provide rigorous data on the effectiveness and generalizability of one relatively light-touch implementation strategy in increasing use of autism EBIs and associated outcomes in diverse under resourced public service settings for underrepresented autistic youth.

**Trial registration:**

Mind the Gap: Clinicaltrials.gov Identifier: NCT04972825 (Date registered July 22, 2021); Remaking Recess: Clinicaltrials.gov Identifier: NCT04972838 (Date registered July 22, 2021); Self-Determined Learning Model of Instruction: Clinicaltrials.gov Identifier: NCT04972851 (Date registered July 22, 2021).

## Background

Psychological and educational interventions are primary service options for autism spectrum disorder. While the number of evidence-based interventions (EBIs) defined as practices shown by high-quality research to have meaningful effects on outcomes [1] has increased over the past decade, most have not been implemented widely with fidelity in the community [2–4]. In particular, families from traditionally marginalized and disenfranchised groups often experience systemic challenges accessing these interventions [5]. Similarly, researchers have not been very successful or deliberate in recruiting representative samples for intervention studies [6]. The Autism Intervention Research Network for Behavioral Health (AIRB) has led the field in diversifying the pool of research participants, reducing barriers to participation, and conducting randomized controlled, multi-site interventions in the community settings where these individuals receive care. Over the last 11 years, we have conducted many randomized controlled trials with families living below or near the federal poverty line and those from diverse racial, ethnic, and linguistic backgrounds. Our community partnered participatory research (CPPR) approach [7] has been critical to AIRB’s success. We engaged community partners (e.g., public schools, community health agencies, parent organizations, autistic advocacy organizations) in developing, adapting, and implementing intervention research studies.

The composition of most research samples, which do not include many minoritized populations or those from low socio-economic strata, means that autism research has quite limited knowledge of what works for whom and under what conditions. This lack of diversity has been criticized in autism spectrum disorder (ASD) research and limits our ability to consider interventions as fully evidence-based [5, 8]. In addition to generally relying on predominantly White and middle class or wealthier samples for trials, autism researchers rarely test interventions in the settings in which they hope interventions ultimately will be used, favoring instead the highly controlled environments of the university laboratory or clinic. Autistic children with the fewest resources are less likely to come to a research site and are most likely to receive intervention through their schools. Our AIRB research team has developed authentic relationships with autistic advocates, families, and strong partnerships with community agencies, such as schools and health clinics, which have allowed us to implement interventions in real world settings, teach adults (e.g., parents, educators) strategies for working with autistic children, and partner with parent-peer mentors to help families navigate the service system.

While we have made significant gains in developing novel interventions and testing them in community settings, we have not addressed the challenges of ensuring that these interventions are implemented at scale or that they are sustainable once the study is over. Indeed, the abrupt discontinuation of research interventions upon conclusion of the studies has been a consistent concern raised by our community partners. Through conversations with these community partners and further examination of the existing literature, we have identified several potential barriers to large-scale implementation and sustainment. First, ASD EBIs often comprise complex, multifaceted packages that require significant training and resources to implement. Often the EBI manual provides no direction for adapting interventions to the context or individual child, family, or classroom; instead, practitioners are expected to select and combine techniques with limited training or supervision. Second, the manuals provide little or no guidance to organizations regarding how to support training and ongoing supervision or the necessary resources for successful implementation. Third, complex interventions require coordination and leadership from many individuals across different institutions, which has historically been challenging in early intervention and school systems [9, 10]. Successful implementation requires teamwork and a positive implementation climate [11]. For example, practitioners need leaders to set expectations, provide resources, and acknowledge positive performance. Working cohesively together can result in a positive implementation climate, which improves satisfaction and retention, as well as child outcomes [2, 12, 13].

One way to build a stronger implementation climate is to create team cohesion across the organization and build common purpose in implementing the intervention. There is little rigorous research on the most effective and efficient way to identify key people and organizations needed for successful implementation and sustainment [14]. Many implementation efforts use convenience or (indiscriminate or arbitrary) selection when deciding who to include on the implementation team. Social Network Analysis (SNA) offers a scientific method for identifying an implementation team that can promote better connections among implementers and improve implementation quality and associated outcomes [15, 16]. While it has demonstrated success in the implementation of other interventions, SNA has not yet been applied to implementing ASD EBIs.

An additional challenge to successful implementation is limited access to high quality training for programs and providers who do not have easy access to training facilities [17]. Often practitioners in rural or low resource areas often cannot access expert training, coaching, consultation, or feedback. Advances in technology and greater access to the internet create opportunity for distance training and implementation support. This technology enables providers in remote settings to receive training on how to effectively implement EBIs resulting in improved access to services enhanced quality of care, more efficient service delivery, and decreased financial burdens for historically underserved populations defined as groups that have traditionally been provided with inadequate ASD services [18]. These training approaches are especially timely given the current COVID-19 pandemic and can be used to scale up training for community providers.

Our community partners often highlight the potential benefit of ASD-specific interventions for other children with NDD. One tension is the inclusion and exclusion of certain participants despite potential benefit to all as well as the need for training in multiple interventions for different populations. Because many children with ASD have co-morbidities, such as intellectual and language impairment, genetic syndromes and mental health concerns, ASD EBIs can be a fit for these other populations. Future research must include a more diverse sample of research participants from infancy through adolescence to understand for whom different interventions work. This would empower community agencies to provide evidence-based intervention to all children and families they serve regardless of identified diagnosis or co-morbidities.

### Theoretical framework and approach

The theoretical framework for our implementation strategy is based on social network and organizational theories. Social network theories related to program implementation prioritize how social connections impact program needs assessment, design, implementation, sustainment and monitoring [19, 20]. Implementation progress and sustainment can be enhanced when staff identify implementation leaders as sources of expertise, advice, and support [21, 22]. Ill-informed selection of implementation leaders can interfere with successful implementation and sustainability. For instance, teams may overlook people who have the best expertise due to a lack of knowledge or a lack of recognition by organizational leaders. Using community-identified leaders for implementation improves the effectiveness of EBIs [23, 24]. The use of SNA allows for systematically identifying members of the team who can support and sustain the implementation of the EBI.

Organizational theory suggests that successful implementation is supported by systematic, planned internal “conditions,” including clearly defined roles, collaborative teams, and transparent expectations. Teams that are thoughtfully assembled, develop an implementation plan, assign roles and responsibilities, and carefully track and support implementation and sustainment in all its stages increase the quality and sustainment of implementation efforts [25, 26]. To support the creation of critical organizational “conditions,” we integrated social network implementation theories and collaborative teaming approaches to develop a multifaceted implementation strategy to facilitate implementation and sustainment of EBIs in low-resourced settings: UNITED (Using Novel Implementation Tools for Evidence-based intervention Delivery). The participating agencies and school districts and the research team have long-standing relationships, which provide an ideal natural laboratory in which to rigorously test, on a large scale, an implementation strategy that is effective for a variety of autism interventions, regardless of the setting or the age group of the intended recipient, to increase rapid uptake of effective practices to reach our most vulnerable children. We will draw on our prior work using community-partnered research to collaboratively test the implementation of three evidence-based interventions (Mind the Gap (MTG); Remaking Recess (RR); and Self-Determined Learning Model of Instruction (SDLMI). Specifically, the primary research question of this study is:

1. Will UNITED result in improved provider fidelity (primary outcome) to each EBI in comparison to implementation as usual (IAU)? We expect that provider fidelity to an EBI (MTG, RR, SDLMI) will be higher in groups using UNITED than IAU groups.
  Other research questions include:
2. Will UNITED result in better child or family outcomes of each EBI (Secondary outcomes)? We expect UNITED will result in better family empowerment for MTG, child peer social engagement for RR, and greater adolescent self-determination for SDLMI than IAU groups.
3. Will UNITED result in greater sustainability of an EBI in the community (secondary outcome)? We expect that sustainability as measured by the Stages of Implementation Completion (SIC) will be greater for UNITED groups versus IAU.
4. (Exploratory): Will team cohesion mediate the effect of EBI on provider fidelity? We expect that team cohesion will be greater in the UNITED condition, and team cohesion will mediate the relationship between the EBI and provider fidelity.
5. (Exploratory): Will better implementation climate predict increase in provider fidelity to each EBI? We expect that schools/agencies with better implementation climate will have greater improvement in provider fidelity.

This study will develop and simultaneously test a multifaceted implementation strategy, UNITED, to support the uptake and implementation of three evidence-based autism interventions across different service settings (public schools and community agencies) and across the lifespan (early intervention, school-aged children, adolescence). Because implementation strategies often are not simultaneously tested across evidence-based interventions, this study has the potential to increase the generalizability of our findings. If successful, UNITED could become a much needed, “light touch” implementation strategy to increase widespread use of evidence-based interventions in community settings.

## Methods/Design

We will compare the use and effectiveness of each intervention (MTG, RR, SDLMI) with and without the addition of our implementation strategy, UNITED. In both UNITED and IAU, we will train community practitioners using remote delivery and provide active coaching via Zoom for at least 6 sessions and up to 12 as dictated by each EBI.

### UNITED

**Fig. 1:**
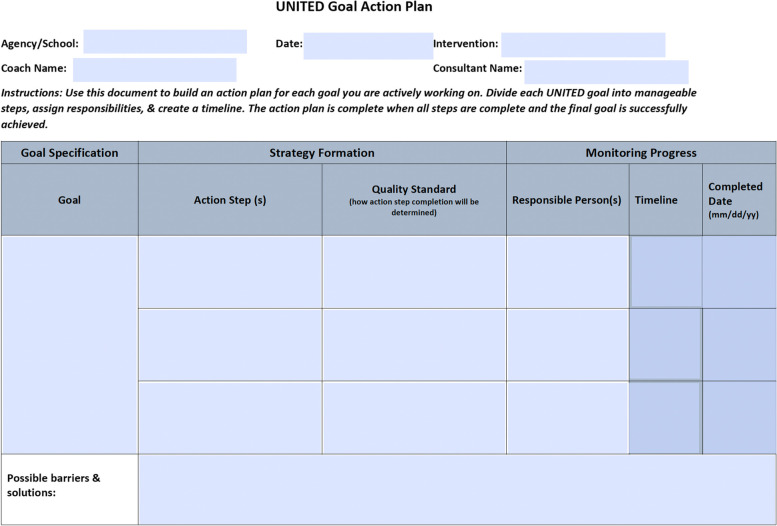
UNITED implementation and sustainment plan template

A facilitator from the research team supports the research site to create a UNITED implementation team that includes leaders (a designated implementation site leader at the agency/school in a leadership role such as an agency director/supervisor or school principal/administrator or other staff such as a peer navigator or teacher) and frontline staff at the community agency/school. The agency/school leader meets with the facilitator via Zoom to review the project & plan how to identify the implementation team. This initial meeting lasts approximately 45 min. During this meeting, the facilitator reviews data from the social network survey with the agency/school leader to determine who will be invited to join the UNITED implementation team. All potential participants will be informed of the time commitment required to serve on the UNITED implementation team at their agency/school. Once nominated and consented, the UNITED implementation team then meets with the facilitator to develop a comprehensive implementation plan of the EBI (one 90-min or two 45-min sessions) that includes goals for active implementation and sustainment. See Fig. 1. During this meeting, the UNITED implementation team assigns tasks and establishes mechanisms for making sure that the action plan stays on track. The UNITED implementation team will meet monthly with the facilitator to review goals and the implementation plan, evaluate success and problem solve as necessary. The monthly check-ins are an opportunity to provide updates, share successes, and discuss challenges that arise. In between each monthly Zoom session, the facilitator checks-in weekly by email or phone with the agency/school leader to provide support and problem solve as needed.

UNITED is premised on the idea that successful implementation in organizations like early intervention systems and public schools requires a team-based approach, in which the team is thoughtfully assembled, develops a plan for implementation, assigns roles and responsibilities, and carefully tracks and supports implementation and sustainment in all its stages. To address these requirements, we combine two well-tested strategies. The first is SNA, a well-validated method for investigating team collaboration and intervention diffusion through graphical analysis. We will use SNA to systematically identify members of the team who support implementation of each EBI. SNA promotes comprehensive team selection by asking staff at different levels of the organizational hierarchy to identify people with the best expertise, rather than relying solely on leaders for team selection, potentially overlooking people with key expertise. Potential implementers at a given site will rank staff regarding who can best support them in using the EBI effectively. This method of building and supporting a team is one of four key types of SNA interventions. During sustainment, we will use SNA to promote UNITED team strengths and potential gaps.

The second is collaborative teaming, which is supported through the strategies such as identifying team structure and roles, development of communication norms and conflict resolution strategies, and development of a clear and operationalized implementation plan. Consultants from the research group assist teams in these collaborative processes. To date, the two strategies that comprise UNITED have not been applied in combination. The UNITED implementation strategy will use SNA to create the most cohesive team at a site (agency/school) that will receive training in collaborative teaming, which in turn will help them collaborate and communicate better about implementation.

### EBIs of Interest

We will pair UNITED with three EBIs that cover the ages of early childhood, childhood, and adolescence. These include Mind the Gap (MTG), a family navigation intervention for children less than 8 years of age who are newly diagnosed with ASD [27], Remaking Recess (RR), a school-based social/peer engagement intervention for children ages 5–12 [3, 4, 28–30], and Self-Determined Learning Model of Instruction (SDMLI), a self-determination intervention for adolescents (13–22 years; 22 is the upper age limit of high school for individuals with disabilities) [31–35].

### Mind the Gap

MTG was developed with a network of stakeholder workgroups to create a system of support to engage under-resourced families in accessing intervention services after an ASD diagnosis. MTG uses a modular approach to family support, which provides opportunities for individualization of the intervention based on each family’s needs and includes coaching from a peer navigator who has been through the process with a family member. Seven modules orient around major topics of interest to families post-diagnosis (e.g., understanding ASD, navigating the system, dealing with stigma, stress management). Each module includes short informational videos, narrated PowerPoint presentations, infographics and brief information sheets, activities, and topic-related worksheets.

### Remaking Recess

RR combines both peer-mediated (employing neurotypical peers to support autistic children) and adult-facilitated (employing school personnel to facilitate social engagement for autistic children) approaches to increase children’s social engagement skills. RR is a school-based social engagement EBI for autistic children that involves direct training and in vivo coaching with educators during recess. RR covers topics such as identifying children’s engagement states with peers, providing common activities and games to scaffold children’s peer engagement, supporting children’s social communication, coaching children through difficult social situations with peers, and providing direct instruction on social engagement skills.

### Self-Determined Learning Model of Instruction

Self-determination is the ability to act as a causal agent in one’s own life in order to set and attain goals. It predicts in-school and post-school success for students with disabilities. Teaching skills associated with self-determination (such as goal-setting, problem-solving, decision-making, self-direction, and other self-regulation and executive abilities) results in enhanced self-determination and more positive student academic and functional outcomes [36]. The SDLMI is an EBI for adolescents with intellectual and developmental disabilities that enables a trained facilitator to teach students to self-direct the goal setting and attainment process to achieve educationally relevant goals and enhance self-determination. SDLMI implementation is guided by adults and consists of a three-phase instructional process that is repeated over time to help students set and attain goals. Each of the three instructional phases presents a problem to be solved by the student in a way that helps the students develop, modify and reach self-selected goals.

### Participants

We will recruit from underserved populations in urban, densely populated areas of 50,000 or more, and rural settings, areas that do not lie inside an urbanized area [37]. We will target autistic children but will include children with other neurodevelopmental disorders (NDD) at the request of the agency/school (see Table 1). MTG will include 48 Peer Navigators working at participating community agencies with peer navigator capacity (e.g. parent support centers; FQHC; community clinics; faith-based agencies) serving autistic children and children with NDD; and 240 families of children age 2–8 (with potential expansion based on community feedback) with ASD or NDD. RR will include 112 elementary school personnel (e.g., playground staff, classroom aides, teachers); and 152 autistic children and children with NDD ages 5–12 (4 per school). SDLMI will enroll 106 school staff serving autistic adolescents and adolescents with NDD; and 252 adolescents, ages 13- 19, who have ASD or NDD (6 per school).

**Table 1:** Procedures & participants across concept studies of Evidence Based Interventions (EBIs)

	**UNITED**	**Implementation as Usual (IAU)**
Procedures across EBIs	Social network analysis to select implementers Collaborative Teaming Training Remote training & coaching in EBI EBI Implementation	Selection as usual of implementers (agency/school selects implementers) Remote training & coaching in EBI EBI Implementation
Mind the Gap (MTG)	10 community agencies 24 peer navigators 120 families with children 2–8 years 12 h of remote didactic training & up to 12 h remote supervision	10 community agencies 24 peer navigators 120 families with children 2–8 years 12 h of remote didactic training & up to 12 h remote supervision
Remaking Recess (RR)	19 schools 56 school personnel 76 autistic children 3 h of remote didactic training/12 h remote coaching RR implementation by practitioners	19 schools 56 school personnel 76 autistic children 3 h of remote didactic training/12 h remote coaching RR implementation by practitioners
Self-Determined Learning Model of Instruction (SDLMI)	21 schools 53 school personnel 126 autistic adolescents 2–4 h of remote didactic facilitator training/up to 12 h remote coaching SDLMI implementation by practitioners	21 schools 53 school personnel 126 autistic adolescents 2–4 h of remote didactic facilitator training/up to 12 h remote coaching SDLMI implementation by practitioners

### Procedures

All Institutional Review Boards (UCLA, UC Davis, University of Rochester, University of Kansas, University of Pennsylvania, Drexel University, and University of Washington) approved this study. Recruitment for MTG in agencies and RR and SDMLI in schools will include an informational email and video about the study and respective intervention and UNITED implementation strategy. If agencies/schools are interested, we then meet with agency leaders/directors or school administrators via videoconference to inform them about the study, participation requirements, and answer any questions (approximately 30 min). Following approval by the agency lead/director or school administrator, we will meet with agency staff or teachers/school staff to obtain their consent to participate in the study. Subsequently, we will ask agency staff or teachers/school staff from each respective intervention to send recruitment materials home to parents/caregivers of autistic individuals under 16 years of age. Recruitment materials explain what is expected of research participants and detail all research activities. If interested, parents/caregivers contact the research team to complete informed consent.

To test UNITED, we will randomize half of the community study sites (agencies for MTG and public schools for RR and SDLMI) to UNITED, and the other half to IAU. We will collect SNA for sites in both study arms. The SNA questions will include identification of agency/school staff to use the EBI and/or provide implementation support. See Measures and Table 2. Randomization will be conducted via a customized system tailored for the research design. The system will make a treatment assignment only if the participating agencies/schools meet study criteria. All randomizations will be further stratified using a minimization allocation method. MTG randomization of agencies will be stratified on the number of peer navigators within the agency (peer navigator ≤ 2 vs. > 2) to ensure that treatment groups are balanced for variables that may correlate highly with outcomes. RR randomization of school teams will be stratified on two baseline measures: (a) number of school personnel who will learn and deliver Remaking Recess (school personnel ≤ 2 vs. > 2), and (b) number of children with ASD/NDD in the school (number of children with ASD/NDD ≤ 2 vs. > 2). SDLMI randomization of school teams will occur within each CRE and will be further stratified on two baseline measures: (a) number of school personnel who will learn and deliver SDLMI (school personnel ≤ 2 vs. > 2); and (b) type of school (middle vs high school).

**Table 2:** Social network questions on the roster questionnaire and the social network survey

Roster Questionnaire (Baseline/T2/T3)				
Q#	Network Question	MTG specific language	RR specific language	SDLMI specific language
1	Name the people who can do the (MTG/RR/SDLMI) intervention at your organization. Please name as many as you can think of.	For example, parents who have older children with disabilities who would be good mentors for parents just learning about their child’s disability.	For example, playground monitors who support inclusion for students with disabilities during recess. Please name as many as you can think of.	For example, special education teachers who support students with disabilities to achieve their goals.
2	Name the people at your organization or outside your organization who can provide implementation support to your staff who are doing the (MTG/RR/SDLMI) intervention. Please name as many as you can think of.	For example, staff who could providing coaching or resource coordination for staff who are doing the intervention with caregivers.	For example, disability specialists, special education teachers, behavioral specialist who could provide coaching or resource coordination. Please name as many as you can think of.	For example, disability specialists, school counselors and behavioral specialist who could provide coaching or resource coordination.
3	Name the people who can make financial decisions for funding the (MTG/RR/SDLMI) intervention at your agency. Please name as many as you can think of.	For example, organizational leaders or administrators.	For example, principals or district leaders.	or example, principals or district leaders
Social Network Survey (Baseline)				
Q#	Network Question	MTG specific language	RR specific language	SDLMI specific language
1	Name the best people at your agency for doing interventions that …	…connect caregivers of child with disabilities to services for the first time post diagnosis. For example, please name parents affiliated with or employed at your agency who have older children with disabilities who would be good mentors for parents just learning about their child’s disability.	…support the social engagement of children with disabilities during recess at your school. For example, staff who are skilled at helping students with disabilities engage with their peers during recess.	… support goal setting and self-determination for students with disabilities For example, special education teachers who are skilled at helping their students with disabilities make goals and achieve them.
2	Name the best people at your agency to support the implementation of interventions that…	…connect caregivers of child with disabilities to services for the first time post diagnosis. For example, please name staff who are skilled at providing support, training, mentorship, resource coordination or agency leadership for the parents who are doing the intervention with caregivers.	…support the social engagement of children with disabilities during recess at your school. For example, staff who are skilled at provide support, training, mentorship, resource coordination or school leadership for staff who are doing recess intervention with students with disabilities.	…support goal setting and self-determination for students with disabilities. For example, staff who are skilled at providing support, training, mentorship, resource coordination or school leadership for staff who are doing the intervention with students.
3	Name the best people at your agency for making financial decisions to support funding for interventions that…	…connect caregivers of child with disabilities to services for the first time post diagnosis. For example, agency leaders, financial directors or organizational administrators who are skilled at securing funding for interventions that support caregivers to connect their children with disabilities to services for the first time post diagnosis.	…support the social engagement of children with disabilities during recess at your school For example, school leaders, financial directors or district administrators who are skilled at securing funding for interventions that support recess intervention for students with disabilities.	…support goal setting and self-determination for students with disabilities. For example, school leaders, financial directors or district administrators who are skilled at securing funding for interventions that support goal setting and self-determination for students with disabilities at your school.
4	Name all the people at your agency you collaborated with during the past year to support interventions for…	…connect caregivers of child with disabilities to services for the first time post diagnosis.	…support the social engagement of children with disabilities during recess at your school.	…support goal setting and self-determination for students with disabilities.
4	Collaborators can include: -people you worked with to provide the intervention -people who provided encouragement, training or guidance about the intervention -People who provided resources needed to do the interventions -people who worked with you to support staff who were implementing the intervention -people you coordinated with who secured funding for the intervention			
Social Network Survey (T2/T3)				
Q#	Network Question			
1	Name all the people at your agency you collaborated with during the past year to support the (MTG/RR/SDLMI) intervention.			
1	For example, collaborators can include: -people you worked with to provide the intervention -people who provided encouragement, training or guidance about the intervention -People who provided resources needed to do the interventions -people who worked with you to support staff who were implementing the intervention -people you coordinated with who secured funding for the intervention			

Experts in each EBI will provide training regardless of randomization condition. MTG targets peer navigators (parents of children with disabilities) who will complete the 12- hour remote training on MTG modules and associated topics (e.g., boundaries, family and child safety, data collection). The research team will conduct the initial 12 h of training and active coaching/check ins once per month over a minimum of 6 and maximum of 12 months. Identified families connected to the community agency will be matched with one peer navigator who will then guide and support the caregiver through completion of the MTG modules with active coaching of the family for up to 12 sessions over 4–6 months. Family needs and preferences will guide topic selection. Active coaching will occur via Zoom, or over the phone, based on family preference. RR targets school staff on the recess yard who supervise all students, including those with ASD/NDD. Training in RR will be remote, all schools will receive a 60-to-90-min didactic training during district professional development. Active coaching (via Zoom) will occur weekly with staff at school for a minimum of 6 and up to 12 sessions during RR implementation with enrolled students. In prior studies, various school personnel (e.g., teachers, counselors, bus attendants, noontime aides, classroom assistants, and one-to-one paraeducators) have served as intervention agents. SDLMI targets school-based providers across grade levels (i.e., 6th – 12th grade) and disciplines (e.g., special education, general education). They will receive 2–4 h of remote professional development in SDLMI. Coaching will take place based on the SDLMI Facilitator Objectives that provide a road map for the facilitator. In school contexts, SDLMI instruction is implemented over the course of a semester (approximately 12 weeks) and then repeated with new goals in subsequent semesters. Practitioners will receive 12 h of remote coaching during sessions with students. We will track any unintended events (i.e., harms, consequences) as reported by site implementers. Participants will receive $25 per data collection time point (baseline, exit, and sustainment).

### Measures

#### UNITED Team Selection

##### *Roster questionnaire*

Organizational leaders will complete a roster questionnaire to identify the people at their site who in the present or future could: (1) do the proposed EBI with the target population; (2) provide implementation support for staff doing the EBI; and/or (3) make financial decisions for funding the EBI. The roster questionnaire will identify who will be included in the selection process of UNITED team members, setting the boundary for project participants at the site [38]. See Table 2.

##### *Social network survey*

People identified in the roster questionnaire will be asked to complete a social network survey where they will identify staff on the roster that would be best for three different dimensions: (1) supporting implementation of the EBI, (2) implementing the EBI and (3) allocating financial support for EBI. See Table 2.

##### *Ranked list*

Using indegree, calculated as the number of people who selected a person divided by the number of people on the roster [39], the top 1–2 people for each dimension will make up the final ranked list of recommended UNITED implementation team members at the agency/school. Most UNITED implementation teams will have two people to support and implement the EBI and one person to allocate financial support for the EBI at the school/agency level; however, some UNITED implementation teams also may have one person in the latter role at the district/system level. While ranked lists will be created for UNITED and implementation as usual sites, we will only share the ranked list with UNITED sites to support implementation team selection. To resolve situations where individuals tie with the same score, causing greater than two top scores, network betweenness scores, defined as the fraction of shortest paths that pass through a node (Linton 1979), will be used to decide who will be listed on the final ranked list. Research coordinators will meet with site leaders to select the UNITED team, using the ranked list as a guide. If the on-site leaders wish to deviate from the suggested interventionists, they will allow them to do so for the following reasons. First, local knowledge, a deep understanding of the setting, culture, and dynamics of the agency/school are of paramount value, and we wish to prioritize that. Second, the on-site leaders may be knowledgeable of key dynamics that are unobservable to the research team. For example, the local leaders may know of personality clashes or problems that will hinder implementation. Third, we know that interventions that can be adapted to local needs and conditions are more readily accepted and more effective than those which are “set in stone.” It is important to collaborate and share control with local leads to build buy-in and develop ownership over the EBI; thus, allowing them to adapt is important. We will track and report reasons provided for the deviations.

### Implementation outcomes

#### *Fidelity*

Because we are testing the effect of UNITED on use and sustainment of three EBIs that already have demonstrated effectiveness, our primary outcome measure is providers’ fidelity to each intervention. We will use the established fidelity procedures for each EBI (i.e., MTG, RR, SDLMI).

#### MTG fidelity

The fidelity of peer coach implementation of MTG will be measured through audio-recording and coding 25% of sessions, as selected by a random number generator. Following recording, a member of the research team will code the audio for fidelity using the MTG Fidelity Checklist. The MTG Fidelity Checklist records the process and procedures for each session, goal setting and review, use of MTG materials and a measure of the quality of the interaction.

#### *RR fidelity*

Fidelity (i.e. use and frequency of intervention delivery) will be measured via a 20-item self-report that captures each RR component. Use will be scored “0” for “no” and “1” for “yes” to measure whether educators used each RR component. The proportion of completed steps (completed steps/total number of RR components) will be used for analyses. Frequency of intervention delivery will be coded on a Likert-type scale from “1” (rarely) to “4” (always) for each component of RR that was used. The average quality rating across all intervention components will be used for analysis.

#### *SDLMI fidelity*

The SDLMI Fidelity Measure is completed by a trained observer or coach and targets three dimension of fidelity: adherence, quality of delivery, and participant responsiveness. It is organized into three sections: Part A – Observation Information (provides general information about the observation, including the SDLMI phase and Student Questions targeted—exposure); Part B – SDLMI Lesson Observation (provides quantitative and qualitative information on the teacher’s implementation of SDLMI core components [Student Questions, Teacher Objectives, Educational Support] – adherence, quality of delivery, participant responsiveness); and Part C – Content Observation (provides quantitative and qualitative information on teacher’s integration of SDLMI into content instruction—quality of delivery, participant responsiveness). Scores on items in each section are summed for each dimension and provide an overall fidelity index.

#### *Stages for Implementation Completion* [40] 

We will measure progress through the implementation phases using the Stages of Implementation Completion (SIC) tool, an eight-stage tool of implementation process and milestones with stages paralleling the phases of implementation (pre-implementation [exploration, preparation], implementation, sustainability). We will measure stages of implementation completion for each EBI: 1) engagement; 2) consideration of feasibility; 3) readiness planning; 4) staff hired and introduce training; 5) fidelity monitoring processes in place; 6) services and consultation to services begin; 7) model fidelity and staff competence and adherence tracked; and 8) competency. The research team will work with the agency/school to gather information to complete each stage of the SIC from recruitment through sustainment. Dates where the SIC activity was completed will be entered into the online SIC portal. We will use the implementation completion score from the SIC, along with economic analysis as part of the SIC.

#### *Cost effectiveness of interventions*

We will use the SIC tool to measure cost effectiveness of UNITED within each stage of implementation. We will track direct, indirect and ancillary costs to implement each EBI at each community site. Costs of implementation will be expressed as average cost per SIC stage for each site. However, corrections will be applied for a site that takes longer to move through a SIC stage. Regression techniques will be used to estimate risk adjusted cost functions, which can vary with such factors as the number of clients treated by a program, or the time required to move through each stage of the SIC. The dependent variable of the regression will be the logged implementation cost for each stage for each site.

#### Implementation Climate

We will measure implementation climate using the Implementation Climate Scale [41], an 18-item rating scale that measures employees’ shared perceptions of the policies, practices, procedures, and behaviors that are expected, rewarded, and supported in order to facilitate effective EBI implementation. The Implementation Climate Scale is a psychometrically validated and reliable instrument (α = 0.81–0.91). We will explore implementation climate as a potential mediation variable.

#### *Team collaboration intensity*

At baseline, exit and sustainment, we will measure the density of connections among people identified on the ranked list for each site for both treatment and control. For each site, we will calculate the number of reported EBI collaboration ties divided by the number of possible EBI collaboration ties [39].

#### *Implementation reach*

At exit and sustainment on the social network survey, we ask each person to identify people from the roster that they collaborated with during the past year to support the EBI being implemented at their site. We will measure implementation reach for treatment and control sites by calculating the number of reported EBI collaboration ties divided by the number of people on the roster.

### Child and family outcomes

We will collect data on several secondary outcomes, including child and family outcome data specific to each intervention, reach and acceptability of the intervention.

### MTG. Family Empowerment

We will use the Family Empowerment Scale [42], a 34-item measure that measures empowerment in families with children who have emotional, behavioral, or developmental disorders. The Family Empowerment Scale has three subscales: Family, Service System, and Social Politics. Higher scores are indicative of higher family empowerment.

### RR. Peer Engagement

The Clinical Global Impression [43] is a 7-point scale designed to measure overall severity in the child’s peer engagement from baseline. Improvement scores at end of intervention range from 1 (Very Much Improved) to 4 (Unchanged) to 7 (Very Much Worse).

### SDLMI*.* Self-Determination

We will use the Self-Determination Inventory: Student Report [36], a standardized measure of self-determination that includes 21 items developed to document change in the self-determination of adolescents aged 13 to 22. The assessment is completed online. The Self-Determination Inventory: Student Report was validated with over 4,500 youth with and without disabilities, including ASD.

### Data analysis

We will analyze effectiveness of the UNITED implementation strategy with each intervention by using a generalized linear mixed model to compare outcomes (primary: MTG/RR/SDLMI fidelity; secondary: MTG = Family Empowerment; RR = CGI; SDLMI = student report) between UNITED and IAU from baseline to end of intervention stage.

Power calculation: Sample size for each study was determined based on statistical power to detect a difference in primary outcome (EBI implementation fidelity) contrast, a between group (UNITED vs IAU) mean comparison in change in fidelity from baseline to the end of intervention phase (assuming 80% power, a Type I error rate of 5%, a within-person correlation in implementation fidelity of *r* = 0.36 – based on preliminary data from each intervention, estimated attrition rate of 20% for MTG and 30% for RR and SDLMI, and variance inflation factor (nesting of providers within schools/agencies) of 1.4 (RR) to 1.6 (MTG & SDLMI). For MTG a difference of 15% in fidelity corresponds to a moderate standardized effect size of d = 0.48 in between strategies change in peer navigator fidelity (based on previous data, sd = 31). For RR a difference of 0.2 corresponds to a moderately large standardized effect size of d = 0.7 in between groups change in paraprofessional fidelity (based on previous data, sd = 0.28). This is a minimally clinically significant difference in change: differences less than 0.2 fidelity amount to differences of less than 2 RR strategies in a 10-min unstructured play time. For SDLMI a difference of 15% in fidelity corresponds to a moderately large standardized effect size of d = 0.79 in between strategies change in paraeducator fidelity (based on previous data, sd = 18.77).

## Trial status

All universities as well as each participating school district’s Institutional Review Boards have approved the study procedures. Each intervention is registered in clinical trials (MTG: NCT04972825; RR: NCT04972838; SDLMI: NCT04972851). At the time of submission of this manuscript (December, 2021), we have already enrolled participants (e.g., administrators, educators, providers) from community agencies and public elementary, middle, and high schools for data collection.

## Discussion

There are a growing number of EBIs for autistic individuals, but few are successfully implemented in under resourced communities and with historically marginalized and minoritized families [5, 8]. Conducting research in community-based settings creates opportunities to build capacity in existing service systems to ensure EBIs are readily accessible, relatively low-cost, and sustainable. In order to have a lasting impact, autism implementation research must focus on broadening access, expanding reach, and facilitating widespread availability of services in the community. Using effective implementation strategies is critical to support providers and organizations use EBIs. The proposed research activities will develop and simultaneously test a multifaceted implementation strategy to support the uptake and implementation of three EBIs that focus on early intervention, school-aged children, and adolescents with ASD with the goal of promoting access and services for underrepresented and under resourced communities. While all implementation strategies are designed to reduce the research-to-practice gap, most strategies are tested with one intervention and are not necessarily designed and tested to support implementation of multiple interventions concurrently. By applying this implementation strategy across our three EBIs, we increase the potential impact and generalizability of our findings. If beneficial, UNITED could be a much needed, “light touch” and low-cost implementation strategy to increase widespread use of EBIs in community settings. Additional research is needed to study the cost-effectiveness of UNITED in both high-resourced and low-resourced settings.

Each EBI has potential for high impact and research advancement. All three studies will be situated in schools or community agencies and will fully transfer the EBI and progress monitoring to the real-world team. Remote delivery of staff training has potential for expanding reach and increasing participation and engagement particularly during COVID-19 restrictions and social distancing requirements.

### MTG

Improving uptake of early intervention for low-resource racial and ethnic minority families in urban and rural communities is essential to improving outcomes for autistic children who may experience intersectional service access disparities based on both their disability and other marginalized identities. MTG improves partnership with the service system, and by working with community agencies, such as Family Resource/Empowerment Centers, we can provide an implementation model for scaling up peer navigator programs to increase access to quality services.

### RR

Recognizing the role that school personnel can play in children’s treatment plans represents an economical way to address the needs of autistic children and their families (who often have to participate in significant and costly out of school treatments). If successful, school personnel will be able to deliver RR with fidelity to autistic children on the playground, and school-based teams will be able to systematically monitor progress, troubleshoot, and support its continued implementation without external support.

### SDLMI

Autistic adolescents from marginalized groups and low-income households are at a higher risk for poor post-school outcomes (e.g., under-employment, limited college enrollment) when compared to their white, higher resourced counterparts. SDLMI studies that extend to autistic adolescents from under-resourced families and communities are needed, and remote methods for training staff may extend reach and participation. This research could provide an evidence-based and sustainable intervention that could improve the adult outcomes of a vulnerable group of children and families.

### Limitations

This is one of the first studies to concurrently test a multifaceted implementation strategy across public service systems (community agencies, public schools), EBIs (MTG, RR, and SDLMI), and life span (birth to young adulthood) for a particular population (autistic individuals). Due to COVID-19, we replaced all observer-rated instruments (e.g., fidelity) with self-rated assessments, and the entire study will be completed remotely (e.g., online consent and surveys, videoconference coaching sessions, etc.). While both practical and necessary, self-report data may introduce bias. However, given the need to understand the providers’ implementation of each EBI in the contexts in which autistic individuals are served, providers’ perspectives will provide pivotal information for the field. Although remote EBI coaching allows us to reach more agencies and schools beyond driving distance to each university, we note that remote EBI coaching introduces additional barriers to implementation (e.g., understanding the service setting, intervention context, end-users, targeted population, etc.) as well as may lead to higher attrition.

## Data Availability

The application described in this manuscript is freely available. Please contact the senior author for more information.

## Abbreviations

AIRB: Autism Intervention Research Network on Behavioral Health
ASD: Autism spectrum disorder
CPPR: Community Partnered Participatory Research
EBI: Evidence-based intervention
IAU: Implementation as Usual
MTG: Mind the Gap
NDD: Neurodevelopmental Disorder
RR: Remaking Recess
SDLMI: Self-Determined Learning Model of Instruction
SIC: Stages of Implementation Completion
SNA: Social Network Analysis
UNITED: Using Novel Implementation Tools for Evidence-based Intervention Delivery
